# Precision Enhanced Bioactivity Prediction of Tyrosine Kinase Inhibitors by Integrating Deep Learning and Molecular Fingerprints Towards Cost-Effective and Targeted Cancer Therapy

**DOI:** 10.3390/ph18070975

**Published:** 2025-06-28

**Authors:** Fatma Hilal Yagin, Yasin Gormez, Cemil Colak, Abdulmohsen Algarni, Fahaid Al-Hashem, Luca Paolo Ardigò

**Affiliations:** 1 Department of Biostatistics, Faculty of Medicine, Malatya Turgut Ozal University, 44210 Malatya, Turkey; 2 Department of Management Information Systems, Faculty of Economics and Administrative Sciences, Sivas Cumhuriyet University, 58140 Sivas, Turkey; yasingormez@cumhuriyet.edu.tr; 3 Department of Biostatistics, and Medical Informatics, Faculty of Medicine, Inonu University, 44280 Malatya, Turkey; cemil.colak@inonu.edu.tr; 4 Department of Computer Science, King Khalid University, Abha 61421, Saudi Arabia; a.algarni@kku.edu.sa; 5 Department of Physiology, College of Medicine, King Khalid University, Abha 61421, Saudi Arabia; fahaid999@yahoo.com; 6 Department of Teacher Education, NLA University College, Linstows Gate 3, 0166 Oslo, Norway

**Keywords:** tyrosine kinase inhibitors, deep learning, bioactivity modeling, cheminformatics-based drug screening, precision oncology

## Abstract

**Background and Objective:** Dysregulated tyrosine kinase signaling is a central driver of tumorigenesis, metastasis, and therapeutic resistance. While tyrosine kinase inhibitors (TKIs) have revolutionized targeted cancer treatment, identifying compounds with optimal bioactivity remains a critical bottleneck. This study presents a robust machine learning framework—leveraging deep artificial neural networks (dANNs), convolutional neural networks (CNNs), and structural molecular fingerprints—to accurately predict TKI bioactivity, ultimately accelerating the preclinical phase of drug development. **Methods:** A curated dataset of 28,314 small molecules from the ChEMBL database targeting 11 tyrosine kinases was analyzed. Using Morgan fingerprints and physicochemical descriptors (e.g., molecular weight, LogP, hydrogen bonding), ten supervised models, including dANN, SVM, CatBoost, and CNN, were trained and optimized through a randomized hyperparameter search. Model performance was evaluated using F1-score, ROC–AUC, precision–recall curves, and log loss. **Results:** SVM achieved the highest F1-score (87.9%) and accuracy (85.1%), while dANNs yielded the lowest log loss (0.25096), indicating superior probabilistic reliability. CatBoost excelled in ROC–AUC and precision–recall metrics. The integration of Morgan fingerprints significantly improved bioactivity prediction across all models by enhancing structural feature recognition. **Conclusions:** This work highlights the transformative role of machine learning—particularly dANNs and SVM—in rational drug discovery. By enabling accurate bioactivity prediction, our model pipeline can effectively reduce experimental burden, optimize compound selection, and support personalized cancer treatment design. The proposed framework advances kinase inhibitor screening pipelines and provides a scalable foundation for translational applications in precision oncology. By enabling early identification of bioactive compounds with favorable pharmacological profiles, the results of this study may support more efficient candidate selection for clinical drug development, particularly in regards to cancer therapy and kinase-associated disorders.

## 1. Introduction

The medical pursuit of tyrosine kinases because of their vital regulatory functions has led to significant research because these enzymes control multiple cellular processes, from signal transduction to cell division and differentiation. Cellular homeostasis depends heavily on these enzymes, which direct the function of signaling networks vital to normal physiology. The deregulated functioning of these enzymes has emerged as a key factor in multiple medical conditions, particularly cancer onset and cancer spread throughout the body. Their dual capability to maintain cellular functions and propel pathological signals demonstrates why protein kinases make excellent therapeutic targets for precision medicine [1,2].

Research has extensively studied tyrosine kinases because their unregulated activation generates cancer pathogenesis through cellular proliferation, impaired apoptosis, and metastasis amplification. The discovery of small molecule inhibitors has provided doctors with a targeted method to disrupt cancer-fueling dysregulated signaling pathways by selectively targeting enzyme dysfunction. Such inhibitors enable personalized treatment through individual patient molecular assessments, while reducing systemic toxicity because of their specific interaction characteristics [3,4]. Beyond oncology, tyrosine kinases regulate neurodegenerative diseases (e.g., Alzheimer’s) and autoimmune disorders (e.g., rheumatoid arthritis), as evidenced by recent advances in kinase-targeted therapies. These enzymes control several conditions, including autoimmune diseases, inflammatory diseases, and metabolic disorders; thus, their therapeutic modulation shows great promise in these contexts [5]. This research utilizes computational and cheminformatics methodologies to analyze tyrosine kinase inhibitors’ (TKIs) therapeutic potential. This research employs structure–activity relationship (SAR) analysis to evaluate chemical interactions while creating bioactivity profiles, which boosts potential therapeutic agent discovery acceleration procedures. This work establishes an interdisciplinary framework by combining machine learning algorithms with deep learning techniques to unite bioinformatics and medicinal chemistry while enhancing discoveries in drug development and personalized medicine [6,7].

Small molecules of tyrosine kinase inhibitors have revolutionized cancer therapy by creating an extremely effective way to halt malfunctioning signaling pathways that promote tumor development and spread. Engineered small molecule inhibitors show outstanding selectivity toward tyrosine kinases in abnormal states, which act to reduce general toxicities together with the effects on unrelated targets. Such precise binding capability enhances treatment outcomes while delivering enhanced quality of life to patients receiving therapy [3]. Precision medicine significantly benefits from tyrosine kinase’s matchless specificity because these enzymes enable treatments that target unique patient molecular and genetic information. Such a precise treatment strategy maximizes therapeutic outcomes and minimizes unwanted side effects through personalization. Due to their ability to manage different cellular processes, tyrosine kinases demonstrate extended therapeutic applications beyond those involved in cancer medicine. The broad effectiveness range of tyrosine enzymes extends into numerous pathological states that span autoimmune disorders, metabolic syndromes, and neurodegenerative diseases, endowing targeted kinase therapy with even broader applications [4]. Researchers have explored the pharmacologic inhibition of tyrosine kinase (TKI) to create combination therapies. Combining TKIs with current immunological and chemotherapy treatments generates synergistic benefits that enhance patient outcomes. High-throughput screening combined with computational modeling technologies simplifies the discovery and optimization of novel TKIs thus speeding the movement between laboratory studies into medical trials. Combining therapeutic innovation with digital methods demonstrates the transformative medical value of tyrosine kinase inhibitors [6,7,8].

The use of sophisticated machine learning methodologies, including convolutional neural networks (CNNs), deep artificial neural networks (dANNs), and conventional machine learning methods, like random forest (RF) and XGBoost, highlights the interdisciplinary strategy employed in this research [9,10]. Most procedures developed in drug discovery proceed slowly, cost a lot, and involve testing thousands of substances, with uncertain success. Advances in AI and dANNs are allowing this field to develop faster, better, and in more innovative ways. AI and dANNs have made a significant impact on drug discovery. Large chemical, biological, and clinical datasets are quickly analyzed by AI, which precisely identifies potential new drugs. AI, mainly dANNs, can prepare computer models that predict how compounds affect their selected targets, reducing early research required for drug development. The key advantages are as follows: the time needed for AI screening is usually shorter than years; with predictive models, laboratories spend less on tests because they target potential successes; they reduce the risk of major errors toward the end of the process; and AI brings forward new chemical possibilities, offering developers novel treatments. Stokes et al. (2020) applied dANNs to identify an antibiotic that had been neglected using standard methods [6]. Atomwise and Insilico Medicine platforms are helping to speed up the development of drugs for cancer and neurodegenerative diseases. AI and dANNs have sped up drug discovery, made it less expensive, increased accuracy levels, and introduced new types of research [6].

AI and deep learning methods are implemented in this work to accurately predict whether tyrosine kinase inhibitors, which target crucial targets in cancer, display the desired bioactivity. With the application of advanced models such as dANNs to a large dataset, this study is able to identify potential drug candidates with superb accuracy. Combining the Morgan fingerprints with fine-tuned hyperparameter values proves our results with the required accuracy and reliability. Using this method, new cancer treatments are developed faster, requiring less time and lower costs than old drug screening processes. In addition to oncology, this new framework can greatly advance drug discovery in many other medical fields. This study seeks to expedite the identification and optimization of tyrosine kinase inhibitors by using cheminformatics and computational techniques, thereby enhancing the field of targeted cancer therapy. This study distinguishes itself by systematically comparing the bioactivity prediction performance of ten machine learning models using an extensive feature representation strategy, filling a methodological gap in kinase-targeted computational drug discovery.

## 2. Results

The dataset used in this study consists of simplified molecular input line entry system (canonical SMILES) strings and pIC_50_ values for each molecule. Canonical SMILES is a format that represents the structure of a chemical compound as a short text string in a specific order. It encodes atomic bonds and structural features using a series of symbols, allowing the structure of molecules to be expressed in plain text. pIC_50_ is a value that quantifies the biological activity of a compound, particularly for molecules interacting with biological targets, such as enzyme inhibitors or receptor binders. In the first stage of the experiment, canonical SMILES strings were converted into RDKIT molecules using an open-source library. Following this, Morgan fingerprints were generated for each RDKIT molecule, completing the feature extraction process. In this study, pIC_50_ values were used as class variables. Samples with a pIC_50_ value below 6 were labeled as class 0, while those with a pIC_50_ value of 6 or above were labeled as class 1. While calculating the Morgan fingerprint values, the minimum and maximum path lengths were set to 1 and 7, respectively. The fingerprint vector size was set to 2048, and the minimum fingerprint size was set to 128. The parameters for considering hydrogen atoms, including branched paths, and accounting for bond order were all set to True. No additional feature selection technique was applied. All 2048-bit Morgan fingerprints and computed physicochemical descriptors were used as input features for all models. After feature extraction and class variable assignment, 20% of the samples were randomly selected to form the testing dataset, whereas 10% were selected for the validation dataset, and the remaining samples (70%) were used for training. The validation dataset was employed for hyperparameter optimization and callback function evaluation in neural network-based models; the training dataset was used to train the models; and the testing dataset was used to evaluate the performance of the trained models. The final descriptor matrix included 21,441 samples and 2052 features (2048 Morgan fingerprint bits + 4 physicochemical descriptors). After stratified splitting, the training set contained 15,008 × 2052 entries, the validation set 2144 × 2052, and the test set 4289 × 2052. The distribution of samples across the classes after segmentation is shown in Table 1. As indicated in Table 1, the number of instances in class 1 is approximately 1.5 times that in class 0. This ratio between the class distributions was maintained throughout the dataset segmentation process for the training, validation, and testing datasets.

After the dataset segmentation phase, each classification method was developed using libraries in Python 3.9. Specifically, Keras was employed to develop the dANN and CNN models, CatBoost and XGBoost were implemented using their respective Python libraries, and the scikit-learn library was used for the remaining methods. Some hyperparameters for each model were then optimized using a random search approach. During the optimization phase, the validation dataset was used for testing, and the optimization process was aimed at maximizing the accuracy metric. The name, type, search space, and optimal values for the hyperparameters of each model are presented in Table 2. Hyperparameters not specified in Table 2 for the relevant models were kept at their default values as provided by the respective libraries.

Table 2 presents the list of hyperparameters optimized for each model and the corresponding ranges specified in the hyperparameter search space. For the dANN and CNN models, the “number of neurons” and “number of filters” hyperparameters contain multiple values due to the depth of these models, which include multiple layers. The definitions of the hyperparameters in this table are as follows:

- n_estimators: Specifies the number of trees or base learners used in the model.
- eta: A shrinkage factor that controls the learning rate at each boosting step.
- max_depth: Defines the maximum depth of trees in tree-based models.
- colsample_bylevel: Sets the fraction of features randomly selected at each tree level (depth).
- learning_rate: Determines the extent to which the model’s weights are updated at each step.
- max_samples: The proportion of data samples randomly selected for each base learner in bagging.
- max_features: Specifies the maximum number of features considered when splitting a node or building a tree.
- max_iter: The maximum number of training iterations the model performs.
- max_leaf_nodes: Sets the upper limit on the number of leaf (terminal) nodes in a decision tree.
- min_samples_leaf: The minimum number of samples required to form a leaf node.
- l2_regularization: The L2 regularization (ridge) penalty applied to model weights to prevent overfitting.
- tol (tolerance): The minimum required improvement in the optimization objective for training to continue.
- C (regularization): Inverse of regularization strength; smaller values specify stronger L1 or L2 regularization.
- min_samples_split: The minimum number of samples required to split an internal node.
- min_split_loss (gamma): The minimum loss reduction required to make a further split in tree-based models.
- min_child_weight: The minimum sum of instance weights (hessian) needed in a child node.
- Number of hidden layers (dANN): The total number of hidden layers in a deep artificial neural network.
- Number of neurons: The number of neurons (processing units) in a given layer.
- Number of CNN layers: The number of convolutional layers in a convolutional neural network.
- Number of filters: Specifies how many filters (kernels) are used in each convolutional layer.
- Number of neurons in Dense: Defines the number of neurons in the fully connected (dense) layer.

Each value in these hyperparameters corresponds to the number of neurons or filters in the respective layers. After completing the hyperparameter optimization process, each model was trained using the optimum hyperparameters and the training dataset. During the training phase, the number of epochs for the dANN and CNN models was set to 500, and two callback functions, lr_callback and early_stopping_callback, were incorporated. The lr_callback function reduces the learning rate by half if no improvement is observed in the validation dataset for two consecutive epochs. The early_stopping_callback terminates training if no improvement is observed in the validation dataset for six consecutive epochs. The performance metrics of the trained models, calculated after completing the training process, are presented in Table 3.

**Figure 1:**
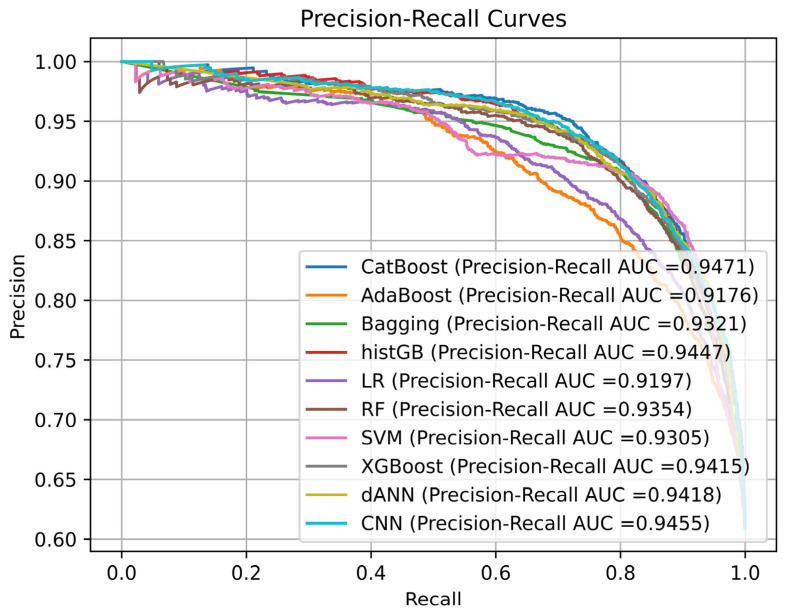
Precision–recall curves for all models.

The accuracy metric is widely recognized as inadequate for evaluating model performance on imbalanced datasets, as it does not provide a fully reliable measure. Instead, alternative metrics are recommended, particularly when a significant class imbalance exists. As shown in Table 3, performance scores for the trained models were calculated using various metrics. A low log loss score is desirable for high model performance, while higher scores in other metrics indicate better outcomes. In this evaluation, the SVM model achieved the highest performance regarding accuracy and F1-score, the dANN model excelled in regards to precision and log loss score, the RF model performed best in regards to recall, and the CatBoost model achieved the best result for the ROC–AUC score. No single model achieved the best results across all metrics; however, the SVM and dANN models showed the best performance for two metrics, while the RF and CatBoost models excelled in for metric each. The remaining models—AdaBoost, Bagging, histGB, LR, XGBoost, and CNN—did not achieve the best result in any metric. Based on the results in Table 3, the SVM model is considered the most effective overall, as it achieved the highest F1-score, which is particularly suitable for imbalanced datasets due to its consideration of the harmonic mean of precision and recall. Reliability is also a crucial factor in model evaluation, alongside accuracy. The log loss score, which quantifies the divergence of predicted probabilities from the actual classes, is an important measure of model reliability. Among the models, the dANN achieved the lowest log loss score and the highest precision, making it the most reliable model based on these results. In addition to these metrics, evaluating model performance on a per-class basis is essential. To achieve this, precision–recall (PR) curves, which are more reliable than ROC curves for imbalanced datasets, were plotted for each model. PR curves of all classes are shown in Figure 1.

Analysis of Figure 1 reveals that the CatBoost model exhibited the highest area under the precision–recall curve score, solidifying its position as the top-performing model based on this metric. This finding aligns with the ROC analysis, where CatBoost also demonstrated superior performance. While all models achieved comparable results, the AdaBoost and LR models displayed inferior precision–recall curves. Among the SVM and dANN models, which consistently demonstrated strong performance across other metrics, the dANN model exhibited a slight edge, suggesting that it is the most robust and effective model based on the average performance across all evaluation metrics.

**Figure 2:**
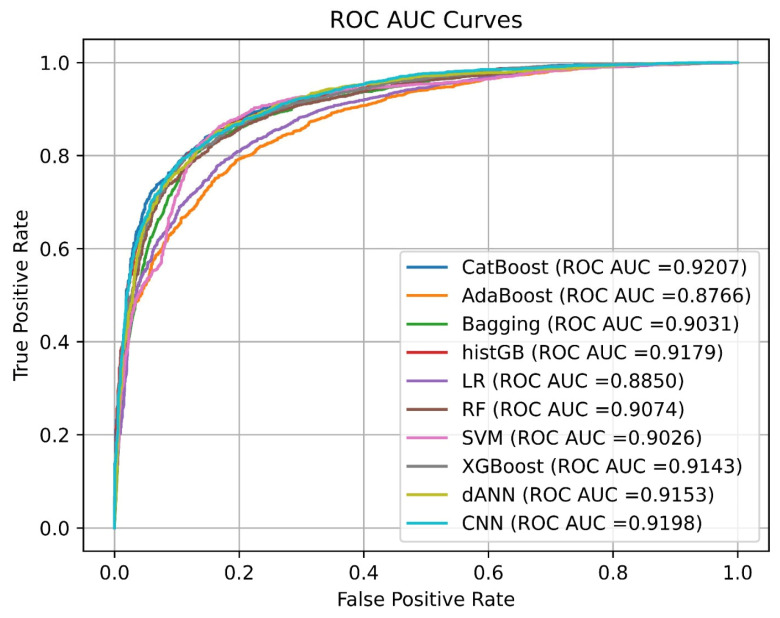
ROC–AUC curves for all models.

Figure 2 depicts the ROC–AUC curves for all models. To compare performance, the ROC curves display the classification outcomes for ten machine learning models. During performance evaluation, CatBoost achieved the best outcomes, with an AUC value of 0.9207, while CNN reached a value of 0.9198 and histGB showed a value of 0.9179. Gradient boosting methods (CatBoost, XGBoost, histGB) and deep learning approaches (CNN, dANNs) provide stable high performing results, although the simpler logistic regression (0.8850) and AdaBoost (0.8766) approaches deliver acceptable outcomes. The discrimination capability of all models remains strong, as shown by AUC values surpassing 0.87, and they display similar patterns in the first part of the false-positive rate spectrum (0.0–0.2).

## 3. Discussion

This article thoroughly reviews the prediction models that aim to investigate the bioactivity and drug-like features of small molecules that target tyrosine kinases. It does this using a robust dataset and advanced computational techniques. Advanced machine learning algorithms underscore the potential of computational techniques to transform contemporary drug discovery processes. This research primarily focuses on the application of CNNs. CNNs are very good at pulling out hierarchical and complex features [11] from molecular data. These features make it easier to accurately model the complex interactions between molecular structure and bioactivity, which could speed up the development of new treatments [12].

Each analytical model provided different strengths, which benefit the current research results. SVMs demonstrated superior F1 scores because these methods prove exceptional at dealing with cases involving strongly imbalanced datasets. Such conditions require the same attention to precision and recall accuracy. The dANN model performed the best by delivering the minimum possible log loss scores. The CatBoost algorithm demonstrated exceptional versatility by achieving the highest rates in both ROC–AUC and precision–recall metrics, indicating its usefulness for dealing with complex datasets with many dimensions [13]. During this research, we employed a random search to optimize the models’ performance characteristics, while performing hyperparameter optimization. By implementing this approach, the developed models achieved high precision and adaptation to the unique statistical characteristics of the underlying dataset. Bioactivity predictions received added robustness by implementing Morgan fingerprints as a molecular structure representation system [14,15].

To select the most suitable model, we used a multi-metric evaluation strategy considering F1-score for imbalanced classification, LogLoss for probabilistic reliability, and ROC–AUC for discrimination capability. Among all models, SVM provided the highest F1-score (87.9%), indicating its superior classification capability. dANN achieved the lowest LogLoss, suggesting it offers the most reliable probabilistic outputs. Thus, the model selection was based on the intended application: SVM for robust classification and dANN for confident candidate selection in downstream biological validation workflows.

Our research demonstrates that machine learning accelerates drug discovery pipelines by leveraging advanced modeling techniques and a comprehensive molecular descriptor dataset, including activity readouts and drug-likeness metrics. An evaluation combining potency assessments with drug-likeness assessments confirmed the safety profile excellence of the identified compounds. Computational approaches demonstrate their potential to cut expensive drug development timelines, according to recent research findings, and they present new opportunities to advance more sophisticated prediction models. This study broadened its predictive model evaluation by testing various frameworks, including traditional algorithms and advanced machine learning approaches, next to CNNs. A detailed assessment approach allowed for a full evaluation of technique features to show each framework design’s advantages and shortcomings. The precise handling of imbalanced datasets by SVMs, combined with CatBoost’s excellence at obtaining high ROC–AUC and precision–recall metrics, indicate its power as a tool for dealing with complex datasets. dANNs exhibited reliable operation because of their low log loss scores, while providing substantial abilities to detect complex data relationships [6,13,16]. The analysis confirmed that dANNs produced the lowest log loss value of 0.25096 compared to that of other evaluated models (Table 3). The dANNs demonstrate superior prediction capabilities, as log loss assessment indicates their ability to penalize several prediction errors, especially when model outcomes are overconfident [17]. The probabilistic prediction precision of SVM (0.37858) and CatBoost (0.34975) are lower than those of other models based on their log loss values. Medical diagnosis and risk assessment applications require accurate probability estimation and classification results because these elements are equally important in generating confidence-aware predictions [18]. The minimization of log loss achieved by dANNs results from its neural network structure, which automatically develops non-linear boundaries while performing gradient-based probability alterations [17]. Furthermore, bioactivity predictions gained crucial power from using Morgan fingerprints as an integral component that extracted detailed structural features. The dANNs utilize these integrated factors to predict bioactivity in tyrosine kinase inhibitors with high accuracy and probabilistic predictions, positioning it as an essential tool for precision medicine development and drug discovery acceleration. While the current models predict bioactivity levels based on molecular features and known kinase targets, direct target engagement (e.g., binding site specificity, off-target interactions) was not explicitly modeled. However, since all molecules were labeled based on experimental pIC_50_ values against specific tyrosine kinases, our approach indirectly ensures relevance to the intended targets. Future studies may incorporate docking simulations or multi-target activity predictions to refine direct targeting capabilities.

Our research builds on and improves previous efforts related to machine learning in finding drugs, focusing on predicting the bioactivity of TKIs. Particularly, a similar study used random forest and support vector machine methods to predict the activity of kinase inhibitor compounds, obtaining an accuracy of around 80% [19]. We attribute our SVM model’s 85.1% accuracy to using Morgan fingerprints and carefully selecting optimal hyperparameters. Another work also studied deep network-based toxicity prediction [16], reporting a log loss of around 0.30. By comparison, our DNN model shows an improved reliability, with a log loss result of 0.25096. 

The comprehensive approach generated essential knowledge points for computational strategy enhancements for bioactivity prediction. The meticulous selection of data points and the addition of molecular descriptors, bioactivity metrics, and drug-likeness scores enhanced the practical value of the predictive models. Cheminformatics integration with modern machine learning ensured that the research accelerated therapeutic candidate identification and then demonstrated how these methodologies scale their potential to enhance pharmaceutical development rates. The results demonstrate the transformative capability arising from using multiple predictive frameworks and advanced datasets in biomedical investigations. The current investigations provide important foundations for next-generation studies that will enhance and expand these methodologies and may involve further modalities and next-generation models like transformers and graph neural networks. Bioactivity forecasting will advance through these enhancements to demonstrate better therapeutic precision during targeted medicine creation [12,20].

Each tested model demonstrated particular benefits, alongside specific restrictions, during evaluation. SVMs demonstrated exceptional performance by reaching the highest F1 score, which makes them optimally suited for working with imbalanced datasets. The results showed that the dANNs model produced the most reliable predictive accuracy by performing with the minimum log loss score. The CatBoost model showed consistent superiority over all other examined models in regards to both precision–recall and ROC–AUC measurement criteria, establishing its position as a flexible framework for processing sophisticated datasets. A combination of various evaluation metrics remains essential when assessing dataset-imbalance because it enables a complete understanding of how well the models perform. A principal strength of this experiment showcases an extensive hyperparameter optimization method through random search optimization for model enhancement. The adapted methodology produced customized models that recognize distinct properties within the dataset to improve predictive accuracy. Incorporating Morgan fingerprints for molecular representation strengthened the model reliability because the methodology effectively retrieves crucial structural drug activity markers. For translational purposes, this research demonstrates how machine learning technology rapidly speeds up the search for tyrosine kinase inhibitors. The models provide predictive information about bioactivity profiles and drug-likeness properties to help researchers select compounds for laboratory testing, thereby reducing drug development costs through more efficient methodology. Detecting toxicity and drug-likeness metrics provides the identified compounds with effective development potential due to their safety and required drug characteristics [7,13,15,16].

Beyond their well-characterized roles in cancer biology, tyrosine kinases have emerged as pivotal regulators in a diverse spectrum of non-malignant diseases—including neurodegenerative, autoimmune, metabolic, and infectious disorders—underscoring their broad therapeutic relevance. Tyrosine kinases, particularly Fyn, have additionally been implicated in the pathogenesis of Alzheimer’s and Parkinson’s diseases. Fyn, a non-receptor tyrosine kinase, plays a critical role in neurodegenerative processes such as synaptic dysfunction and neuroinflammation, which are hallmarks of both Alzheimer’s and Parkinson’s diseases. Fyn is activated by amyloid-beta (Aβ) oligomers, which have been linked to the pathogenesis of Alzheimer’s disease (AD) through their interaction with both Aβ and tau proteins [21]. Increased expression of the FynT isoform is associated with neurofibrillary degeneration and reactive astrocytosis in AD, suggesting its involvement in cognitive impairment [22,23]. Fyn’s signaling pathways play a role in synaptic plasticity and neuronal differentiation, making it a potential therapeutic target for AD [24,25]. Similar to its function in AD, Fyn expression is upregulated in Parkinson’s disease dementia (PDD) and Lewy body dementias and is associated with cognitive decline and tau pathology. The dysregulation of Fyn may contribute to neuroinflammation and tauopathy, which are critical to the progression of Parkinson’s disease [22]. Janus kinases (JAKs) inhibit the JAK-signal transducer and transcription (STAT) signaling pathway, reducing inflammation by blocking cytokine signaling. They are effective in the management of rheumatoid arthritis (RA) and psoriasis and offer an alternative to conventional therapies such as biologics and disease-modifying antirheumatic drugs (DMARDs) [26]. Clinical studies have shown that JAKis, including tofacitinib and baricitinib, significantly improve disease activity and patient quality of life in RA and psoriasis [27,28]. Retention rates for JAKis are higher when used as a monotherapy or first-line therapy [29].

The findings of this research also open avenues for future studies. Better model prediction results might be achieved by implementing additional data modalities, including proteomics and transcriptomics. By implementing graph neural networks or transformer models, researchers can improve their predictive abilities due to these architectures’ better understanding of complex data relationships. XAI techniques and their development may provide deeper insight into the predictive factors while enhancing the trust and transparency within computational drug discovery methods.

This study contributes to the field by offering a comprehensive evaluation of machine learning and deep learning models for kinase inhibitor bioactivity prediction using an integrated feature set that includes both Morgan fingerprints and physicochemical descriptors. The methodological novelty stems from the comparative analysis across a wide range of models—ensemble, shallow, and deep learning—alongside rigorous hyperparameter optimization. Additionally, the inclusion of log loss as a metric for probabilistic reliability has rarely been highlighted in previous TKI-related studies, positioning this work as a valuable methodological contribution to the application of AI in computational pharmacology. This study focuses primarily on predicting molecular bioactivity rather than on pharmacokinetic behaviors such as drug delivery or tissue-specific distribution. Drug targeting was addressed implicitly through kinase-specific datasets, but delivery mechanisms remain outside the current scope of this study and represent a promising area for future integration using systems pharmacology or nanocarrier modeling.

## 4. Materials and Methods

### 4.1. Dataset Description

This research utilized the 28,314 small compounds from the ChEMBL database, including bioactivity, toxicity, and drug-likeness information targeting tyrosine kinases. Tyrosine kinases serve as essential therapeutic targets against cancer because these enzymes control multiple cellular operations, spanning signal transduction to cell division and differentiation. The dataset focuses on the following specific tyrosine kinase proteins:

- ABL (Abelson tyrosine-protein kinase 1): 1970 compounds;
- EGFR (epidermal growth factor receptor): 9508 compounds;
- PDGFR (platelet-derived growth factor receptor): 1750 compounds;
- FGFR (fibroblast growth factor receptor): 3156 compounds;
- MET (hepatocyte growth factor receptor): 3950 compounds;
- VEGFR (vascular endothelial growth factor receptor): 1211 compounds;
- KIT (stem cell factor receptor): 1692 compounds;
- RET (rearranged during transfection): 896 compounds;
- JAK (Janus kinase): 4203 compounds;
- ALK (anaplastic lymphoma kinase): 2087 compounds;
- SRC: 3846 compounds.

The curated dataset facilitates the development of predictive models for the bioactivity and drug-like properties of small molecules targeting tyrosine kinases. This dataset is particularly valuable for machine and deep learning applications to explore structure–activity relationships, assess drug-likeness, and predict the potential therapeutic efficacy of tyrosine kinase inhibitors [30].

### 4.2. Data Processing

The dataset includes molecular identifiers, canonical SMILES (simplified molecular input line entry system) and bioactivity measures such as IC_50_ and pIC_50_ values. Bioactivity thresholds were applied to classify compounds as active (IC_50_ ≤ 1000 nM), inactive (IC_50_ ≥ 10,000 nM), or intermediate (1000 nM < IC_50_ < 10,000 nM). Compounds with missing or zero IC_50_ values and repeated SMILES entries were excluded from the dataset [30].

### 4.3. Molecular and Bioactivity Descriptors

The RDKit library calculated four molecular descriptors, i.e., molecular weight (MW), LogP and hydrogen bond donors (NumHDonors), and acceptor (NumHAcceptors) values. The toxicity and drug-likeness scores were evaluated through DataWarrior software 6.1.0, while pIC_50_ values were generated for all compounds to create standardized bioactivity representations [30]. Although essential molecular descriptors (e.g., LogP, hydrogen bond donors/acceptors, MW) were included, additional ADMET (absorption, distribution, metabolism, excretion, and toxicity) features were not directly modeled. These remain crucial for holistic drug design and will be considered in future extensions of this framework.

### 4.4. Methods

This study employed ten distinct methods to predict the bioactivity and drug-like properties of small compounds targeting a tyrosine kinase protein. Among these, a deep learning-based CNN model was developed and compared against other approaches, including dANN, CatBoost, AdaBoost, Bagging, histGB, LR, RF, SVM, and XGBoost. The hyperparameter optimization for each model was conducted using the random search technique. The selection of machine learning and deep learning algorithms in this study was guided by their distinct capabilities in handling the complexities of tyrosine kinase inhibitor bioactivity prediction. dANNs and CNNs were chosen for their ability to learn the hierarchical representations of molecular structures, which is essential for capturing non-linear relationships between chemical features and bioactivity. These models excel at processing high-dimensional fingerprint data and identifying subtle structural patterns that influence kinase binding. For ensemble methods, CatBoost and XGBoost were employed due to their robustness in handling imbalanced datasets, a common challenge in bioactivity classification, as well as their native support for categorical feature encoding and gradient-based optimization. SVMs were included for their effectiveness in high-dimensional spaces, leveraging margin maximization to improve generalization of fingerprint-based input data. Random forest and histGB were selected for their ability to handle heterogeneous features and provide interpretable feature importance metrics, which aid in understanding the molecular determinants of bioactivity. LR served as a baseline model due to its simplicity and interpretability, while AdaBoost and bagging were utilized to assess the impact of boosting and aggregation techniques on predictive performance. The molecular descriptors, including Morgan fingerprints, molecular weight, and partition coefficient (LogP), were prioritized because they encode critical structural and physicochemical properties relevant to drug–target interactions. Morgan fingerprints capture the circular substructures that often correspond to pharmacophoric features essential for kinase inhibition, while descriptors like LogP and hydrogen bond counts align with drug-likeness criteria, ensuring the practical relevance of the predicted bioactive compounds. Together, these methods and descriptors provide a comprehensive framework for TKI bioactivity prediction, balancing predictive accuracy, computational efficiency, and interpretability.

Morgan fingerprints, or extended-connectivity fingerprints (ECFP), were used to appropriately depict molecular structures for machine learning. This feature encoding technique encodes the local chemical environment of atoms in the molecules into fixed-length binary vectors, encapsulating substructural information at different radii. The fingerprints functioned as input features for all models, facilitating the efficient extraction of significant patterns associated with bioactivity and drug-like qualities [19].

### 4.5. Morgan Fingerprints

Morgan fingerprints are a widely used method for representing molecular structures as binary vectors, which are used in cheminformatics for tasks such as identifying molecular similarity and classification. These fingerprints are generated by iteratively considering the neighborhood of each atom in a molecule, with the neighborhood defined by bonds and atom types within a specified radius. The algorithm works by starting from each atom and recursively expanding the neighborhood, generating a unique identifier for each atom based on its local environment. This process produces a series of bits representing the presence or absence of specific molecular substructures. One of the key features of Morgan fingerprints is the ability to handle cyclic, branched, and complex structures, which are commonly encountered in drug design and materials science. The radius of the neighborhood considered can be adjusted, typically ranging from one to three, to capture varying levels of structural detail. Morgan fingerprints are also flexible, as they can be encoded into vectors of varying lengths, depending on the desired resolution of the molecular representation. These fingerprints are particularly useful for tasks such as virtual screening, quantitative structure–activity relationship (QSAR) modeling, and similarity searches. They have been widely adopted in drug discovery due to their efficiency and ability to encode structural information in a compact and interpretable format. Moreover, the fingerprinting process is computationally efficient, making it suitable for large-scale analysis of molecular databases [31].

### 4.6. Classification Models

#### 4.6.1. Convolutional Neural Network

**Figure 3:**
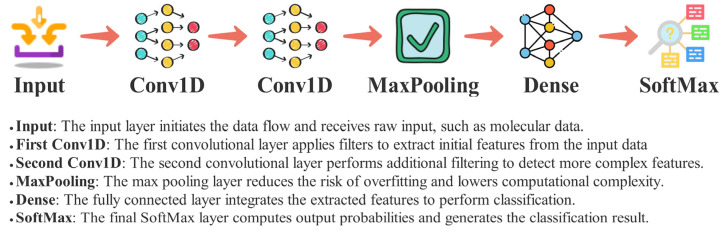
Proposed convolutional neural network architecture.

CNNs are deep learning models specifically designed for processing grid-like data structures, such as images or sequences [32]. A CNN comprises multiple layers, including convolutional, pooling, and fully connected layers, which extract hierarchical features from the input data. The convolutional layers apply learnable filters (kernels) that perform spatial convolutions, enabling the detection of local patterns such as edges, textures, or shapes [33]. These filters are shared across the input space, reducing the number of parameters and enhancing generalization. Pooling layers, often using operations like max pooling or average pooling, downsample feature maps, reducing computational complexity and providing spatial invariance [34]. Fully connected layers at the network’s end aggregate extracted features to make predictions, such as classifying input data. The hierarchical nature of CNNs allows them to learn increasingly complex representations, from the low-level features in the initial layers to the high-level semantic features in deeper layers. Modern advancements, including architectures like ResNet [11] and EfficientNet [10], have significantly enhanced the performance and efficiency of CNNs by introducing innovations like skip connections and compound scaling. Despite their success, CNNs can require substantial computational resources and large datasets for training, which has driven research to develop optimization techniques and more efficient architecture. In CNN-based models, various architectural structures can enhance performance and adapt to specific tasks. The architecture of the CNN model utilized in this study is illustrated in Figure 3.

The architecture presented in Figure 3 illustrates a CNN model with a depth of two, defined by the number of Conv1D layers included in the architecture. By varying the number of Conv1D layers in CNN models, the depth of the model can be adjusted. Since Figure 3 illustrates a model with two different depths, the Conv1D layer is depicted multiple times to reflect this. In the proposed CNN model, the number of Conv1D layers and filters in each layer were optimized to enhance performance. As the dataset used in this study is one-dimensional, Conv1D layers were employed to process the data effectively. For each Conv1D layer, the kernel size was set to 5, and the padding parameter was configured as “same” to preserve the input dimensions. The activation function was set to ReLU for all layers except the final layer, where a SoftMax activation function was applied. The Adam optimizer was utilized for training, and binary cross-entropy was selected as the loss function. As shown in Figure 3, the model begins with an input layer, where the data is initially received. The data is then passed through a series of CNN layers. Following the convolutional layers, a MaxPooling layer is incorporated to help reduce the risk of overfitting. In the final stage, the output is processed through a fully connected layer, and the model is completed with another fully connected layer followed by a SoftMax layer for classification.

#### 4.6.2. Classification Algorithms Used in the Study

dANNs, CatBoost, AdaBoost, Bagging, histGB, LR, RF, SVM, and XGBoost algorithms were used to build the classification models. dANNs consist of multiple layers that enable hierarchical feature learning. They are trained via backpropagation and gradient descent, effectively modeling complex, non-linear patterns. Despite challenges like vanishing gradients, they have shown high performance in regards to image recognition, NLP, and time series analysis and are widely used in fields such as healthcare and finance [34]. CatBoost is a gradient boosting algorithm optimized for categorical data. It uses ordered boosting to prevent target leakage and encodes categorical variables during training, reducing overfitting. With features like oblivious trees and GPU support, CatBoost delivers high performance for tasks such as classification and ranking, especially in domains like finance and healthcare [13,35,36]. AdaBoost builds a strong classifier by iteratively combining weak learners, often decision stumps. It increases weights on misclassified instances, directing future learners’ focus. While effective in reducing bias and variance, it is sensitive to noise. Applications include text classification and medical diagnosis [37,38,39]. Bagging enhances model stability by training multiple models on bootstrapped data subsets and aggregating their predictions. It reduces variance and overfitting, particularly in high-variance models like decision trees. Its robustness and parallelizability make it suitable for finance, healthcare, and image analysis tasks [40]. histGB accelerates training by binning continuous features, reducing the cost of split finding. It supports large, high-dimensional datasets with techniques like feature bundling. Implementations like LightGBM are used in fast, scalable applications across finance and e-commerce [41]. LR is a simple and interpretable model for binary classification. It uses the logistic function to model the probability of outcomes based on input features. While limited in handling non-linearity, regularization improves its performance. LR is widely applied in medical and business analytics [42]. RF is an ensemble method that aggregates multiple decision trees trained on bootstrapped data. By introducing feature randomness, it reduces overfitting and improves generalization. It is scalable, interpretable, and used extensively in finance, bioinformatics, and environmental modeling [43,44]. SVMs find the optimal hyperplane to separate classes, maximizing the margin. Kernel functions enable the handling of non-linear data. Known for robustness in high-dimensional spaces, SVMs are applied in text classification, image recognition, and bioinformatics, although training can be computationally demanding [45]. XGBoost is a high-performance gradient boosting algorithm known for its speed and accuracy. It incorporates regularization, second-order optimization, and efficient tree-splitting, making it suitable for large-scale classification and regression tasks. Its widespread use spans finance, healthcare, and e-commerce [46,47,48].

### 4.7. Validation Metrics

In QSAR modeling, the evaluation of predictive performance requires the use of appropriate statistical metrics that reflect both classification effectiveness and probabilistic reliability. In this study, we employed a comprehensive set of validation metrics to assess the performance of all machine learning and deep learning models. The primary metrics used were accuracy, precision, recall, F1-score, area under the ROC–AUC, area under the PR–AUC, and log loss. Accuracy measures the proportion of correctly predicted instances over the total number of instances, but it may be misleading in imbalanced datasets. Precision refers to the proportion of true positives among all positive predictions, indicating model reliability in identifying active compounds. Recall (sensitivity) measures the ability of the model to detect all relevant instances (i.e., active compounds). F1-score is the harmonic mean of precision and recall, providing a balanced measure especially important for imbalanced datasets. ROC–AUC evaluates the trade-off between true positive and false positive rates across various thresholds, reflecting overall discrimination capability. PR–AUC is particularly suitable for imbalanced datasets, emphasizing model performance on the minority class (usually, the active compounds). Log loss quantifies the accuracy of probabilistic predictions, penalizing confident but incorrect predictions. Lower log loss indicates better-calibrated probability outputs. These metrics were calculated on the test dataset, which was kept separate from training and validation processes, to ensure unbiased performance evaluation. The combined use of these metrics allows for a robust and multidimensional understanding of model quality, which is essential in QSAR modeling where both classification accuracy and prediction confidence are critical.

## 5. Conclusions

In this study, various machine-learning models were used to predict the bioactivity of tyrosine kinase inhibitors, and their performances were evaluated. The best results were obtained with SVM and dANN models. The SVM model showed the highest performance, with 85.1% accuracy and an 87.9% F1-score. This proves that SVM can work effectively, even on imbalanced datasets. The dANN model provided the most reliable probabilistic predictions, with the lowest log loss value of 0.25096. These findings indicate that machine learning can play an important role in accelerating drug discovery processes and reducing costs. This study helps discover new tyrosine kinase inhibitors by (1) showing that SVM and dANN yield the best results, (2) adding Morgan fingerprints to highlight substructures linked to activity, and (3) using optimization strategies to correct imbalances in the training data. This research shows how to effectively use computer-based methods to speed up kinase drug development by closely comparing the results to those of successful experimental methods. This study’s novelty lies in its integrative use of Morgan fingerprints with a diverse set of conventional and deep learning models, including dANNs and CNNs, to predict the bioactivity of tyrosine kinase inhibitors. Unlike prior studies that relied on a single model or limited descriptors, our comparative framework assessed ten distinct algorithms, with optimized hyperparameters.

## Figures and Tables

**Table 1:** Number of samples in training, testing, and validation datasets in regards to the classes.

Dataset Name	Class 0	Class 1	Total
Training (70%)	5865	9143	15,008
Testing (20%)	1676	2613	4289
Validation (10%)	838	1306	2144
Total	8379	13,062	21,441

**Table 2:** Name, type, and space of the hyperparameters and optimum values for each classification model.

Model Name	Hyperparameter Name	Hyperparameter Type	Hyperparameter Space	Optimum Value
CatBoost	n_estimators	Integer	Low = 250, High = 2000	484
	Eta	Real	Low = 0.0001, High = 0.1	0.04938
	max_depth	Integer	Low = 2, High = 16	7
	colsample_bylevel	Real	Low = 0.0001, High 0.9999	0.77038
Adaboost	learning_rate	Real	Low = 0.001, High = 0.999	0.82553
	n_estimators	Integer	Low = 40, High = 1000	893
Bagging	max_samples	Real	Low = 0.001, High = 0.999	0.56529
	n_estimators	Integer	Low = 5, High = 50	36
	max_features	Real	Low = 0.001, High = 0.999	0.89916
histGB	learning_rate	Real	Low = 0.01, High = 1	0.07531
	max_iter	Integer	Low = 50, High = 1000	478
	max_leaf_nodes	Integer	Low = 4, High = 64	25
	max_depth	Integer	Low = 4, High = 32	23
	min_samples_leaf	Integer	Low = 4, High = 64	40
	l2_regularization	Real	Low = 0.0, High = 0.5	0.00232
LR	Tol	Real	Low = 0.00000001, High = 0.001	0.00037
	C	Real	Low = 0.001, High = 32	29.09220
	max_iter	Integer	Low = 50, High = 1500	54
RF	n_estimators	Integer	Low = 50, High = 1000	813
	max_depth	Integer	Low = 2, High = 10	10
	min_samples_split	Integer	Low = 2, High = 8	2
SVC	C	Real	Low = 0.1, High = 32	4.04035
	Tol	Real	Low = 0.00001, High = 0.01	0.00630
XGBoost	learning_rate	Real	Low = 0.001, High = 0.999	0.06745
	min_split_loss	Integer	Low = 0, High = 15	5
	max_depth	Integer	Low = 3, High = 15	12
	min_child_weight	Integer	Low = 1, High = 8	5
dANN	Number of hidden layers	Integer	Low = 1, High = 15	6
	Number of neurons	Integer	Low = 20, High = 500	69, 429, 207, 428, 57, 57
	learning rate	Real	Low = 0.00001, High = 0.01	0.00153
CNN	Number of CNN layers	Integer	Low = 1, High = 15	5
	Number of filters	Integer	Low = 20, High = 500	74, 128, 94, 138, 145
	Number of neurons in Dense	Integer	Low = 20, High = 500	433
	learning rate	Real	Low = 0.00001, High = 0.01	0.000733

**Table 3:** Performance scores of models using optimum hyperparameters for the testing dataset.

Model Name	Accuracy	Precision	Recall	F1-Score	ROC–AUC Score	Precision–Recall AUC Score	Log Loss Score
CatBoost	84.658%	86.217%	89.055%	87.612%	0.9207	0.9471	0.34975
AdaBoost	79.855%	81.310%	86.912%	84.017%	0.8766	0.9176	0.65290
Bagging	83.702%	85.682%	87.945%	86.798%	0.9031	0.9321	0.53274
histGB	84.309%	85.926%	88.787%	87.332%	0.9178	0.9447	0.35703
LR	81.091%	83.898%	85.343%	84.613%	0.8849	0.9197	0.46362
RF	82.723%	82.432%	91.045%	86.524%	0.9074	0.9354	0.40047
SVM	85.055%	86.627%	89.246%	87.917%	0.9025	0.9305	0.37858
XGBoost	83.702%	85.029%	88.902%	86.922%	0.9143	0.9415	0.36915
dANN	84.402%	87.442%	86.873%	87.156%	0.9152	0.9418	0.25096
CNN	84.355%	86.780%	87.677%	87.226%	0.9198	0.9455	0.42950

## Data Availability

Data is contained within the article and Supplementary Materials.

## Supplementary Materials

The following supporting information can be downloaded at: https://www.mdpi.com/article/10.3390/ph18070975/s1.
